# Overexpression of WDR62 is associated with centrosome amplification in human ovarian cancer

**DOI:** 10.1186/1757-2215-6-55

**Published:** 2013-07-30

**Authors:** Yu Zhang, Yan Tian, Jing-Jing Yu, Jie He, Jia Luo, Sai Zhang, Cen-E Tang, Yi-ming Tao

**Affiliations:** 1 Department of Obstetrics and Gynecology, Xiangya Hospital, Central South University, Changsha, Hunan, China; 2 Department of Surgical Oncology, Hunan Cancer Hospital, The Affiliated Hospital of Xiangya Medical College, Changsha, Hunan, China; 3 Institute of Medical Sciences, Major Human Disease Tissue Bank, Xiangya Hospital, Central South University, Changsha, Hunan, China

**Keywords:** Ovarian cancer, WDR62, Centrosome

## Abstract

**Purpose:**

To assess the clinical significance of WD40 repeat containing 62 (WDR62), a novel centrosome abnormalities-associated gene, in ovarian cancer.

**Materials and methods:**

In this study, WDR62 expression was assessed by western blot (6 ovarian cancer cell lines) and immunohistochemistry (primary epithelial ovarian cancer clinical specimens), and clinical variables were collected by retrospective chart review. Centrosome amplification was assessed by immunofluorescence staining in ovarian cancer cell lines, and by immunohistochemistry staining in ovarian cancer samples.

**Results:**

Six ovarian cancer cell lines exhibited significant WDR62 protein overexpression, and amplification of centrosome. High-grade ovarian cancer specimens exhibited significantly stronger nuclear staining of WDR62 than low-grade ovarian carcinoma specimens (80.4% *vs* 41.3%; *P*<0.012). High WDR62 expression was strongly associated with supernumerary centrosome count in tumor cells (*P* < 0.001).

**Conclusion:**

Our findings suggest that WDR62 overexpression is related to centrosome amplification in ovarian cancer. It may be a novel useful differentiation biomarker and a potential therapy target for OC. Further assessment of WDR62 expression is highly warranted in large, prospective studies.

## Introduction

Epithelial ovarian cancer accounts for about 3% of total cancer cases in women [1]. Ovarian cancer is a heterogeneous disease comprising distinct molecular and consists of different histological types, which can be readily differentiated by histological evaluation [2]. Different histological types of ovarian cancer seem to be regulated by different pathogenic pathways.

As a candidate “hallmark” of cancer cells, centrosome amplification (CA) has been frequently detected in a growing list of human solid and haematological cancers, as well as in pre-neoplastic lesions, including those of the ovarian cancer [3,4]. Evidence suggests that CA is involved in the transition from early to advanced stages of carcinogenesis [5,6]. Deregulated centrosome duplication or maturation often results in increased centrosome size and/or number, both of which show a positive and significant correlation with aneuploidy and chromosomal instability, thus contributing to cancer formation [7,8]. p53 is mutated in 90% of human cancers and has been extensively correlated with aneuploidy, genomic instability, and centrosome amplification [9,10]. Some centrosome-associated proteins, such as Polo-like kinase 1 (PLK1), Aurora-A and transforming acidic coiled-coil 1 (TACC1), are documented to be important during the carcinogenesis of OC [11]. Genes involved in spindle formation, centrosome functions and mRNA transport along the microtubule tracks should provide further information on potential markers of docetaxel resistance [9]. In addition, centrosome abnormalities occurred more frequently in ovarian tumors with high grade and aggressive serous subtype [4]. In light of these findings, identification of centrosome abnormalities-associated proteins may reveal novel biomarker or potential therapeutic targets of OC.

The WD40 repeat containing 62 (WDR62) is a very recently identified centrosome-associated gene, which includes 32 exons, a single CpG island and poly-adenylation signal. WDR62 is located at the human chromosome 19q13.12 region and is synthetic to the regions of CEBPG, GPI and UBA2 genes, which are all verified to play important roles in DNA replication and cell cycle progression [12,13]. WDR62 protein, which mediates cellular signaling, transcription, mitotic and apoptotic functions, has 15 WD40 repeat domains and six potential mitogen-activated protein kinase (MAPK) phosphorylation sites in the C-terminal. Notably, the endogenous expression of WDR62 was strongly accumulated at the spindle poles of dividing cells but not in the nucleus when mitosis occurred, suggesting a critical role of WDR62 in Hela cell proliferation [14]. Some recent studies have also documented a critical role of WDR62 in the proliferation of neuronal precursors, and that mutation of the WDR62 gene will induce microcephaly and dysplasia of human brain [15]. So far, to our knowledge, the role of WDR62 in human malignancies remains unknown.

Given the relationship between WDR62 and centrosome amplification found in various cancers, it is intuitive to hypothesize that WDR62 expression may critically mediate the development of human ovarian cancer. Therefore, we planned the present expanded investigation in ovarian cancer for confirming our preliminary hypothesis.

## Materials and methods

### Tissue specimens

In this study, a total of 85 human ovarian cancer samples and 6 normal ovarian surface epithelium samples were collected after surgery at Xiangya Hospital, Central South University and Hunan Cancer Hospital, the Affiliated Hospital of Xiangya Medical College from 2008 to 2010. Among these 85 cases of OC, fresh specimens of high grade ovarian cancer (n = 46) and low grade (n = 29) were collected for Western blot analysis. In addition, tissues were embedded in paraffin after fixation in 10% formalin for histological diagnosis and immunohistochemistry (IHC) analysis. Diagnosis was verified by a pathology review at the institutional gynecologic oncology tumor board. All of the patients were staged according to the International Federation of Gynecology and Obstetrics (FIGO) surgical staging system [16]. A gynecologic pathologist reviewed all of the pathology results for all of the patients. The detail biomedical factors of these tumor types are summarized in Table 1. Prior informed consent was obtained from all recruited patients for ovarian carcinoma tissue specimens collection and the study protocols were approved by the Ethics Committees of two participating hospitals.

**Table 1:** Association of WDR62 expression and clinical characteristics

	**Cases**	**WDR62 expression**		
		**Negative**	**Positive**	***p *value**
**Age (years)**				
<60	34	14 (41.2%)	20 (58.8%)	0.157
≥60	51	13 (25.5%)	38 (74.5%)	
**Tumor size (cm)**				
<5	25	11 (44.0%)	14 (56.0%)	0.132
≥5	60	16 (26.7%)	44 (73.3%)	
**FIGO grade**				
Low	29	18 (62.1%)	11 (37.9%)	0.0004
High	46	9 (19.6%)	37 (80.4%)	
**FIGO stage**				
I	14	9 (64.3%)	5 (35.7%)	0.006
II	15	8 (53.3%)	7 (46.7%)	
III	18	6 (33.3%)	12 (66.7%)	
IV	28	4 (14.3%)	24 (85.7%)	

### Ovarian cancer cell lines

A2780 cell line was purchased from Institute of Biochemistry and Cell Biology, Chinese Academy of Sciences, Shanghai, China. SKOV3, SW626 Caov-3, OVCAR3 and OV-90cell lines were purchased from American Type Culture Collection (ATCC, Manassas, VA). The cells were cultured in low glucose Dulbecco’s Modified Eagle Media (DMEM, GIBCO, Gaithersburg, MD) and supplemented with 10% fetal bovine serum at 37°C under an atmosphere of 95% air and 5% CO_2_.

### Immunofluorescence (IF) staining

Immunofluorescences staining of centrosomes were performed as previously described [17]. Briefly, treated cells were rinsed with ice-cold phosphate- buffered saline (PBS, pH 7.4) and were fixed with 3% paraformaldehyde. The cells permeabilized with 0.1% Triton X-100 were incubated with 5% bovine serum albumin in PBS containing 0.05% Tween-20 (PBS-T) for 30 min. After washing for three times with PBS-T, phalloidin conjugated with FITC was applied and the nuclei were counterstained by DAPI (Invitrogen). Images were captured using microscope (Nikon Eclipse TE2000-S, Tokyo, Japan) from three independent experiments.

### Western blot

Total protein was extracted and separated by SDS-PAGE under reducing conditions and then transferred onto PVDF membrane (Millipore, Bedford, MA). The blocked membranes were then respectively incubated with the primary antibodies at 4°C overnight followed by HRP-conjugated secondary antibodies (KPL, Gaithersburg, MD. 1:3000 dilution) for 1 hour at 37°C. Bands were visualized using the enhanced chemiluminescence kit (Santa Cruz Biotechnology, Santa Cruz, CA). The target signals were quantified by BandScan software (Bio-Rad Laboratories, Hercules, CA) and defined as the ratio of target protein relative to α-Tubulin.

### Immunohistochemistry

Tissue sections (4 μm thick) were prepared from paraffin-embedded blocks. After antigen retrieval treatment in 10 mM citrate buffer (pH 6.0) at 95°C for 10 min, immunostaining was performed using the Envision System with diaminobenzidine (Dako, Glostrup, Denmark). The tissue sections were stained for WDR62 (dilution 1:400; Sigma-Aldrich) detection using a mouse monoclonal antibody, and a subsequently Streptavidin-Peroxidase system (Zhongshan Goldenbridge Biotechnology, Beijing, China). The negative controls for IHC were carried out under the same experimental conditions by omitting the primary antibody. The semiquantitation for intensity was scored on a scale of 0, negative; 1, weak; 2, moderate and 3, strong [18]. We also evaluated the approximate proportion of cells showing immunoreactive score (IS) (0, < 1%; 1, single to 5%; 2, 6-50%; 3, 51-75%; and 4,>75%) to give information about the relative number of positive cells within the specimen (frequency score) [19]. These two kinds of scores were then multiplied to generate the IS for each tissue specimen. Receive operating characteristic (ROC) curve analysis was employed to assess cutoff score for overexpression of WDR62. The score was selected as the cut-off value, which was closest to the point of maximum Youden’s index (sensitivity + specificity-1) was used for determination of optimal cut-off values of the diagnostic tests. OC case designated as “negative expression” for WDR62 was those with scores below or equal to the cut-off value (IS < 2), while “positive expression” tumors were those with scores above the value (IS ≥ 2).

### Immunohistochemistry for centrosomes

Centrosome structures and count was estimated according to the definition as previously described [17,20]. Briefly, endogenous peroxidase was quenched with 3% H_2_O_2_, slides were blocked with 5% normal goat serum for 15 min, followed by incubation with mouse anti-human g-tubulin antibody to identify centrosomes (200 μg/mL; Santa Cruz, Inc.) for 1h at room temperature. Centrosome structures were identified and enumerated in four randomly selected fields of stained tissue sections using Zeiss axioplan microscope. Cells with more than two centrosomes per cell were counted in 200 consecutive cells and an average calculated among the nine samples in each group. The investigator counting the number of centrosomes was blinded to WDR62 expression.

### Statistical analysis

Statistical analyses were performed using SPSS 13.0 software (SPSS, Chicago, IL, USA). Kruskall–Wallis and Mann–Whitney *U* nonparametric tests were utilized to compare differences between WDR62 expression levels. The correlation between WDR62 expression and clinicopathological features of OC patients was analysed by the *χ2* test or Fisher’s exact test. All the tests were two-sided and *p* < 0.05 was considered as statistically significant.

## Results

### WDR62 expression and centrosome amplification in ovarian cell lines

**Figure 1:**
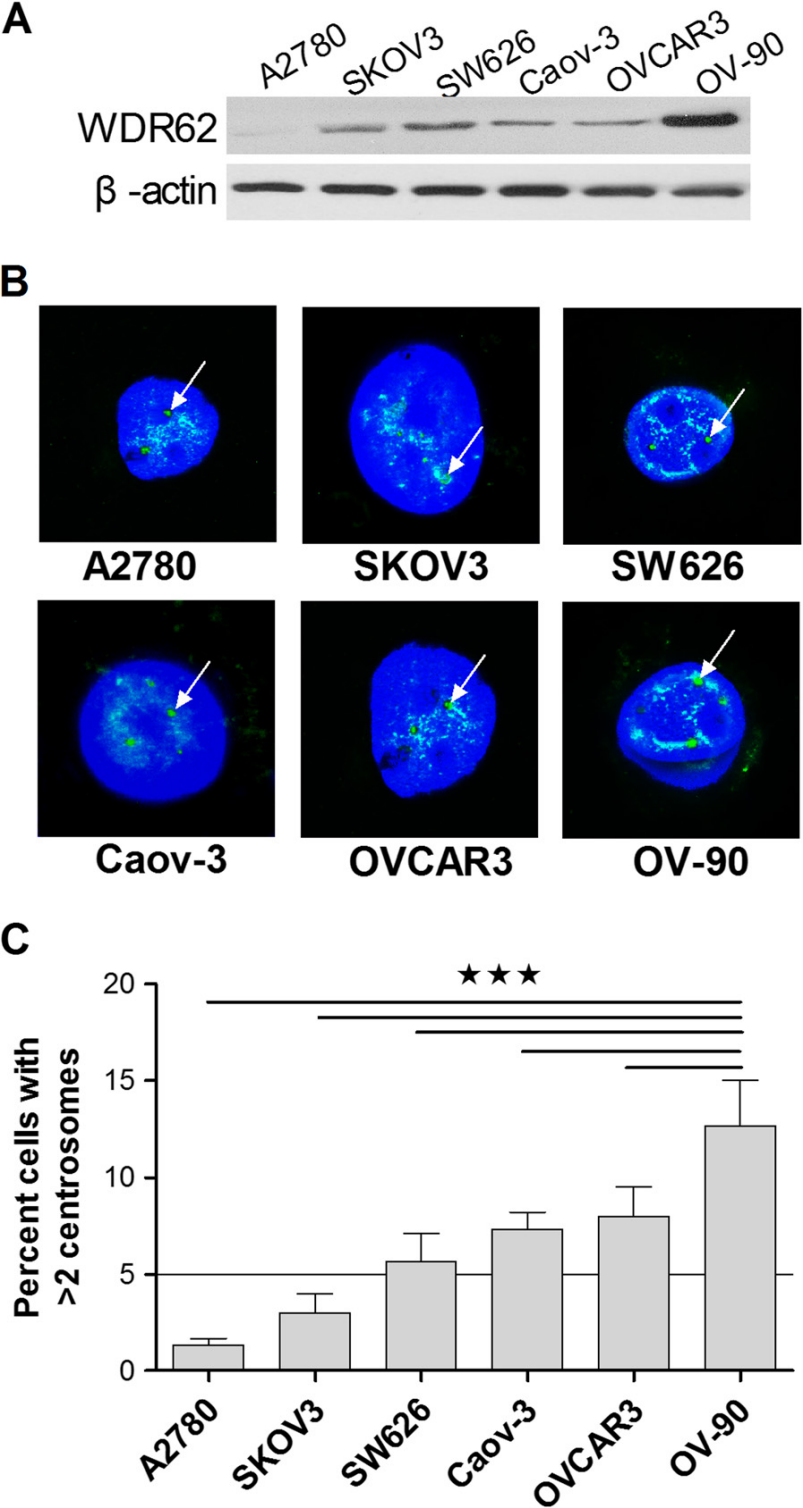
**WDR62 expression in ovarian cancer cell lines. (A)** Whole cell lysates from multiple ovarian cancer cell lines were subjected to Western blot using primary anti-WDR62 antibody. **(B)** Cells in culture was exposed to anti-centrosome antibodies. After secondary antibody-FITC (green), cells were exposed to 4’, 6’-diamidino-2-phenylindole to stain nuclei blue and examined by fluorescent microscopy. White arrows indicated centrosomes. **(C)** After fluorescent staining with anti-centrosome antibodies, the number of cells with more than two centrosomes was counted (at least 400 cells counted) and compared between cell lines.

Western blot analysis was conducted on multiple OC cell lines for WDR62 protein expression. All cancer cell lines expressed high levels of WDR62 protein (Figure 1A), and expression levels in OC cells (SKOV3, SW626, Caov-3, OVCAR3, and OV-90) were significantly higher compared with A2780 cells, which are P53 wild type cell lines. Since WDR62 overexpression is associated with centrosome amplification, these six cell lines were then examined for supernumerary centrosomes. After immunofluorescent staining (Figure 1B), the number of visible centrosomes was counted, and the percentage of cells with more than two centrosomes per cell was calculated (Figure 1C). Cell lines were considered aneuploid if >5% of cells exhibit more than two centrosomes per cell [4], and all ovarian cancer cell lines exhibited supernumerary centrosomes. Interestingly, the WDR62 expression and average centrosome count of high grade ovarian cancer cells (OV-90) were significantly higher than others.

### Expression of WDR62 protein in ovarian cancer tissues

**Figure 2:**
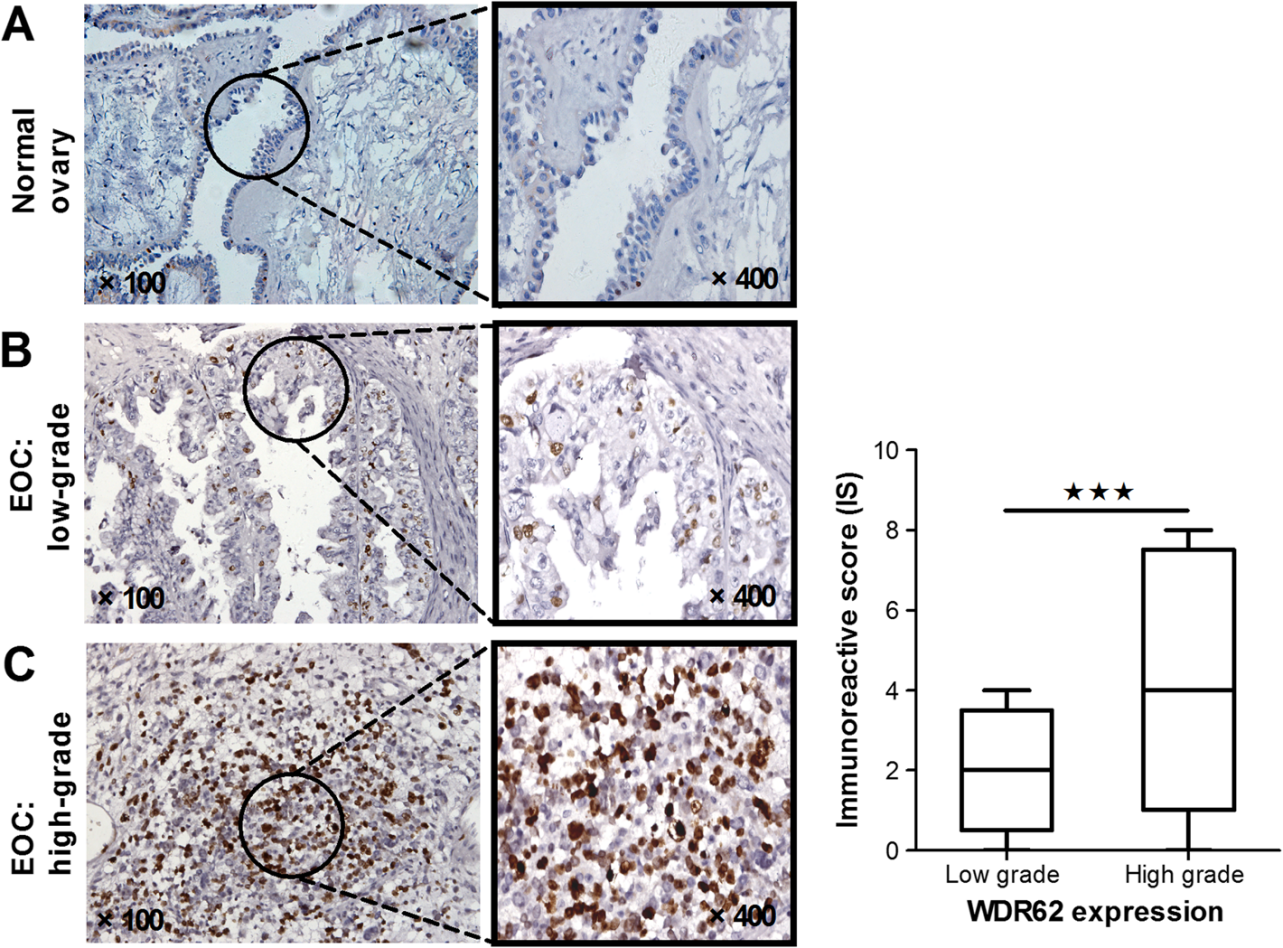
**Immunohistochemistry for WDR62.** Six normal ovaries **(A)** and 85 epithelial ovarian cancer (EOC) specimens were stained for WDR62. Degree of staining in low-grade and high-grade ovarian carcinoma are shown (**B** and **C**, respectively). Original magnification, ×100 (low power) or ×400 (high power).

**Figure 3:**
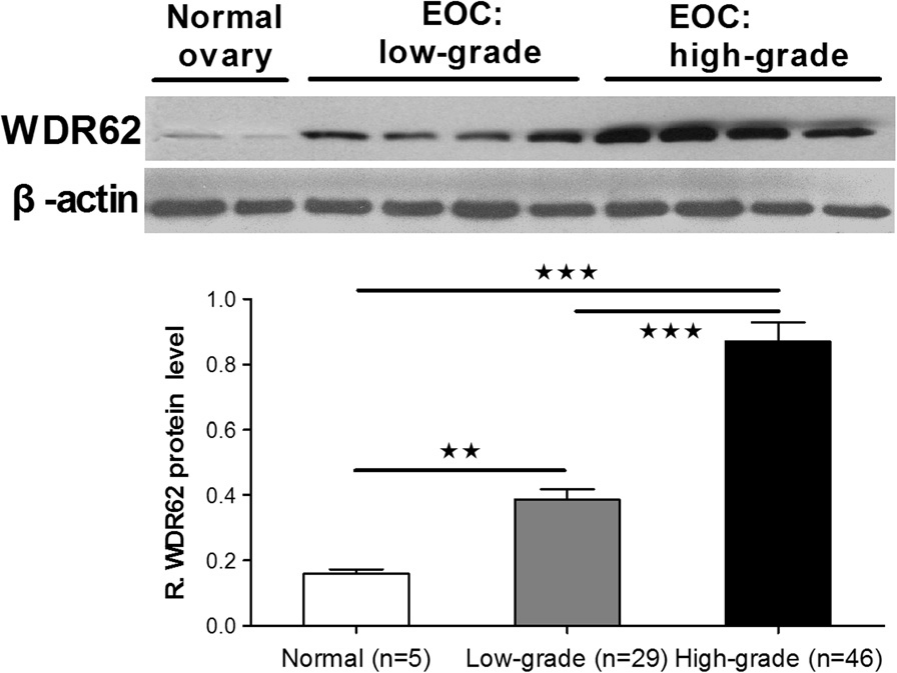
Western blot examination of WDR62 protein in low-grade and high-grade ovarian carcinoma.

Six normal ovarian epithelium samples had shown negatively IHC staining (Figure 2A). As shown in Figure 2B & C. Generally, most high-grade OC showed nuclear staining or only had extremely faint cytoplasmic expression. The low-grade carcinomas had significantly more sections that expressed strong nuclear staining than high-grade carcinomas. WDR62 protein overexpression is associated with clinical features. An analysis of clinical factors and their association with WDR62 expression was conducted (Table 1). WDR62 expression was significantly different when comparing early stage (I-II) and advanced (III-IV) patients. The upregulation of WDR62 was validated by western blot analyses. Protein extracted from high-grade OC showed stronger evidence of WDR62 expression (Figure 3).

### WDR62 overexpression is associated with centrosome amplification in OC

**Figure 4:**
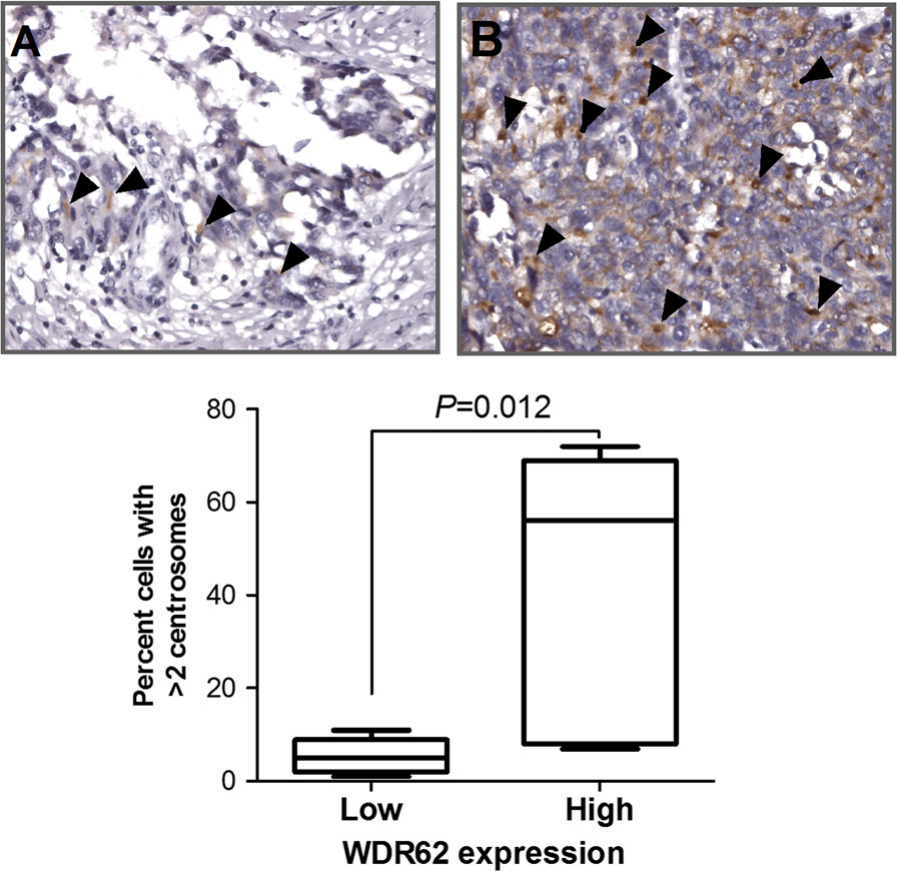
**Centrosome amplification in ovarian cancer samples.** The nine patient samples with the highest WDR62 expression and the nine samples with the lowest expression were subjected to immunohistochemistry against g-tubulin to identify centrosome. Samples with high expression **(A)**, had a significantly higher percentage of cells with supernumerary centrosome than those with lower expression **(B)**. The percentage of cells with more than two centrosomes seen in 200 consecutive cells in each group, with the mean, 25th and 75th quartiles, and minimum and maximum values. Original magnification, ×400 (high power).

Centrosome amplification has been observed in OC cells in which WDR62 overexpression (Figure 1B & C), in the OC tissues with the low and high WDR62 expression (as in Figure 4A & B, respectively). Centrosome amplification was defined as more than two centrosomes in any one cell. In patients with the highest WDR62 expression, the mean number of cells with centrosomal amplification was 56.2% +/− 29.5%, significantly higher than in patients with low expression (11.9%, SD 4.7%; *p* = 0.012).

## Discussion

Although WDR62 expression has been detected in human tissues, such as heart, skeletal muscles, testes and neuronal precursors in brain [14,15], its expression pattern and roles in human malignancies remain to be elucidated. The novel findings from this study are that WDR62 is overexpressed in most of the epithelial ovarian cancer cell lines and tumors especially in high-grade carcinoma of the ovary which was associated with high frequency of p53 mutation [21-23].

In this study, we have shown that the higher WDR62 expression have concurrent significantly higher centrosome count in OC cell lines and tissues. WDR62 overexpression may contribute to carcinogenesis by centrosome amplification, leading to abnormal mitosis. Our results show that WDR62 overexpression was significantly higher in high-grade carcinoma than in other types of ovarian cancer. Centrosome amplification is a mechanism that can lead to chromosomal instability, which in turn, results in further mutations. Therefore, WDR62 overexpression may result in genetic instability that may not only predispose to the rapid development of epithelial ovarian cancer, but also predispose cells to further genetic alterations and heterogeneity with evolving resistance to therapy. Consistent with this, increased Aurora-A kinase expression was strongly related to centrosome amplification and poor prognosis of OC. Aurora-A has also been shown to interact with p53 and reduce its effectiveness as a protector of genome integrity [9,24]. We have described a direct correlation between WDR62 expression and centrosome amplification, which provides a clear pathway towards the development of aneuploidy. WDR62 maybe an effecter of centrosome amplification in OC. We propose that WDR62 overexpression is an important biomarker for the diagnosis and possibly associated with tumorigenesis of ovarian carcinoma.

## Conclusion

Our present study suggests that WDR62 overexpression is an important molecular change specifically related to OC with centrosome amplification, which seems to play a role in both tumor initiation and progression. In addition, WDR62 overexpression can be a potential biomarker for the detection and differentiation grade of OC. Examination of WDR62 targeting agents in OC is warranted.

## Abbreviations

FIGO: International Federation of Gynecology and Obstetrics; IHC: Immunohistochemical; WDR62: WD40 repeat containing 62.

## Competing interests

The authors declare that they have no competing interests.

## Authors’ contributions

Both YZ and YT contributed equally to this work. YZ, YT and YMT conceived the idea, designed and wrote the manuscript. JJY and JH carried out experiment of molecular biology and cell biology, advice on the conception. JL performed clinical data collection and interpretation. SZ performed statistical analysis and interpretation. CET contributed to the conception of the study, reviewed the manuscript. All authors read and approved the final manuscript.
